# Oral colonization by *Levilactobacillus brevis* KABPTM-052 and *Lactiplantibacillus plantarum* KABPTM-051: A Randomized, Double-Blinded, Placebo-Controlled Trial (Pilot Study)

**DOI:** 10.4317/jced.57771

**Published:** 2021-05-01

**Authors:** José Nart, Sara Jiménez-Garrido, Anaïs Ramírez-Sebastià, Erola Astó, David Buj, Pol Huedo, Jordi Espadaler

**Affiliations:** 1 Department of Periodontology, Universitat Internacional de Catalunya, Barcelona, Spain; 2 Nart Dental Clinic, Barcelona, Spain; 3 AB-Biotics SA, Barcelona, Spain

## Abstract

**Background:**

To determine the oral colonization capacity of the strains *Levilactobacillus brevis* KABPTM-052 (CECT 7480) and *Lactiplantibacillus plantarum* KABPTM-051 (CECT 7481) in healthy subjects.

**Material and Methods:**

This randomized, double-blinded, placebo-controlled study included 40 volunteers (22 females, 18 males; age range 18-55 years) with healthy gingiva or mild gingivitis, allocated to receiving probiotic chewing gum (n=20) or placebo (n=20) b.i.d for 6 weeks. At baseline and after 6 weeks of treatment, a periodontics specialist collected saliva samples to assess probiotic colonization by qPCR, and analysed dental plaque, gingival index and dental probing pocket depth in Community Periodontal Index (CPI) teeth subset. Protocol was registered as NCT03540498.

**Results:**

Treatment compliance was high (99%). Both *L. brevis* and *L. plantarum* were detected in the oral microbiota at baseline. After 6 weeks, volunteers receiving probiotic showed a significant increase of both *L. brevis* (*p* = 0.017) and *L. plantarum* (*p* = 0.004) versus placebo. This effect remained significant after adjusting for gender and gingival index at baseline. In the probiotic group, reduction in plaque index significantly correlated to higher levels of *L. brevis* (rho = 0.57, *p* = 0.022) but not of *L. plantarum* at study endpoint, and the number of subjects with dental plaque was reduced during intervention (7 of 17, *p* = 0.016). No such effects were observed in the placebo group. No adverse drug reactions were reported.

**Conclusions:**

*Levilactobacillus brevis* KABPTM-052 and *Lactiplantibacillus plantarum* KABPTM-051 colonize the buccal microbiota of healthy volunteers, and higher colonization by *L. brevis* positively correlated to reduction in dental plaque.

** Key words:**Probiotic, Levilactobacillus brevis, Lactiplantibacillus plantarum, oral colonization, oral microbiota, dental plaque.

## Introduction

Oral diseases such as gingivitis are amongst the most prevalent human diseases and are caused by microbial molecules derived from the accumulation of dental plaque (1,2). The progression of gingivitis may result in periodontitis, a more serious inflammatory disease that may lead to tooth loss (1). Since the ethology of these diseases is clearly polymicrobial (2), antimicrobial therapies may not be effective and, therefore, alternative strategies are required.

One promising preventive strategy relies on the use of probiotics (3-8). Probiotics are live microorganisms which, when administered in adequate amounts, confer a health benefit for the host (9). A number of potential benefits arising from the use of probiotics have been demonstrated, including increased resistance to infectious diseases (10,11), alleviation of lactose intolerance (12), prevention and treatment of various gut diseases (7), prevention and treatment of vaginal and urogenital infections (14), and reduction of serum cholesterol concentration (15,16). Several clinical studies have evaluated the effect of different strains of probiotics on oral health parameters, reporting conflicting results, thus suggesting that some, but not all strains, may exert a beneficial effect (7). Of note, studies have mostly focused on subjects with significant gingival condition, while use of probiotics as preventive treatment in subjects with healthy gingiva or mild gingivitis remains poorly documented.

Desirable traits of probiotics for oral use include demonstrating antimicrobial activity against oral pathogens and displaying a favourable safety profile (e.g. heterofermentative metabolism resulting in reduced acidification, lack of transmissible antibiotic resistance genes). The strains *Levilactobacillus brevis* KABPTM-052 (CECT 7480) and *Lactiplantibacillus plantarum* KABPTM-051 (CECT 7481), now being renamed as *Levilactobacillus brevis* and *Lactoplantibacillus plantarum* (17), have previously shown inhibitory activity against **Porphyromonas gingivalis**, *Treponema denticola* and *Fusobacterium nucleatum* *in vitro*, a low acidogenic activity, lack of transmissible antibiotic resistances genes and good *in vitro* potential for oral colonization (18). However, these strains have been tested in a randomized trial in subjects with gingivitis (5), as well in a randomized trial in subjects with oral pain, but their true capacity to colonize the oral cavity *in vivo* has not been assessed.

To further investigate the health benefits of the strains *L. brevis* KABPTM-052 and *L. plantarum* KABPTM-051 we designed and performed a double-blinded, placebo-controlled study. The aim of this study is to validate the ability of these two strains to colonise the oral tissues and, secondarily, to investigate their potential benefits in subjects with healthy gingiva or mild gingivitis.

## Material and Methods

Forty healthy volunteers within age range 18-55 years old were recruited for the double-blind randomized placebo-controlled clinical trial. The study was carried out in Clínica Universitària d’Odontologia (Universitat Internacional de Catalunya, Barcelona, Spain) during the period October 2016 to February 2017, in accordance with the Declaration of Helsinki, adhered to the CONSORT 2010 statement (www.consort-statement.org). The protocol was approved by the Ethics Committee of Clínica Universitària d’Odontologia (Universitat Internacional de Catalunya, Barcelona, Spain) and the study was registered as NCT03540498 on ClinicalTrials.gov on May 07, 2018.

-Study Population

Healthy non-smoker subjects of both sexes, within the 18-55 years old age range and with at least 20 natural teeth were selected. Exclusion criteria included: pregnant or lactating women; subjects suffering from chronic illness (e.g. diabetes, renal problems, cancer); allergies to the ingredients of the products; antibiotic or probiotic treatment in previous 8 and 4 weeks, respectively; gingival index (GI) ≥ 1.5 (19), dental plaque index (PII) ≥ 2.0 (20), dental pocket depth ≥ 5 mm or more than two untreated caries; subjects under orthodontic treatment; use of bactericidal mouthwashes (e.g. chlorhexidine) in previous 4 weeks.

-Study Design

**Figure 1:**
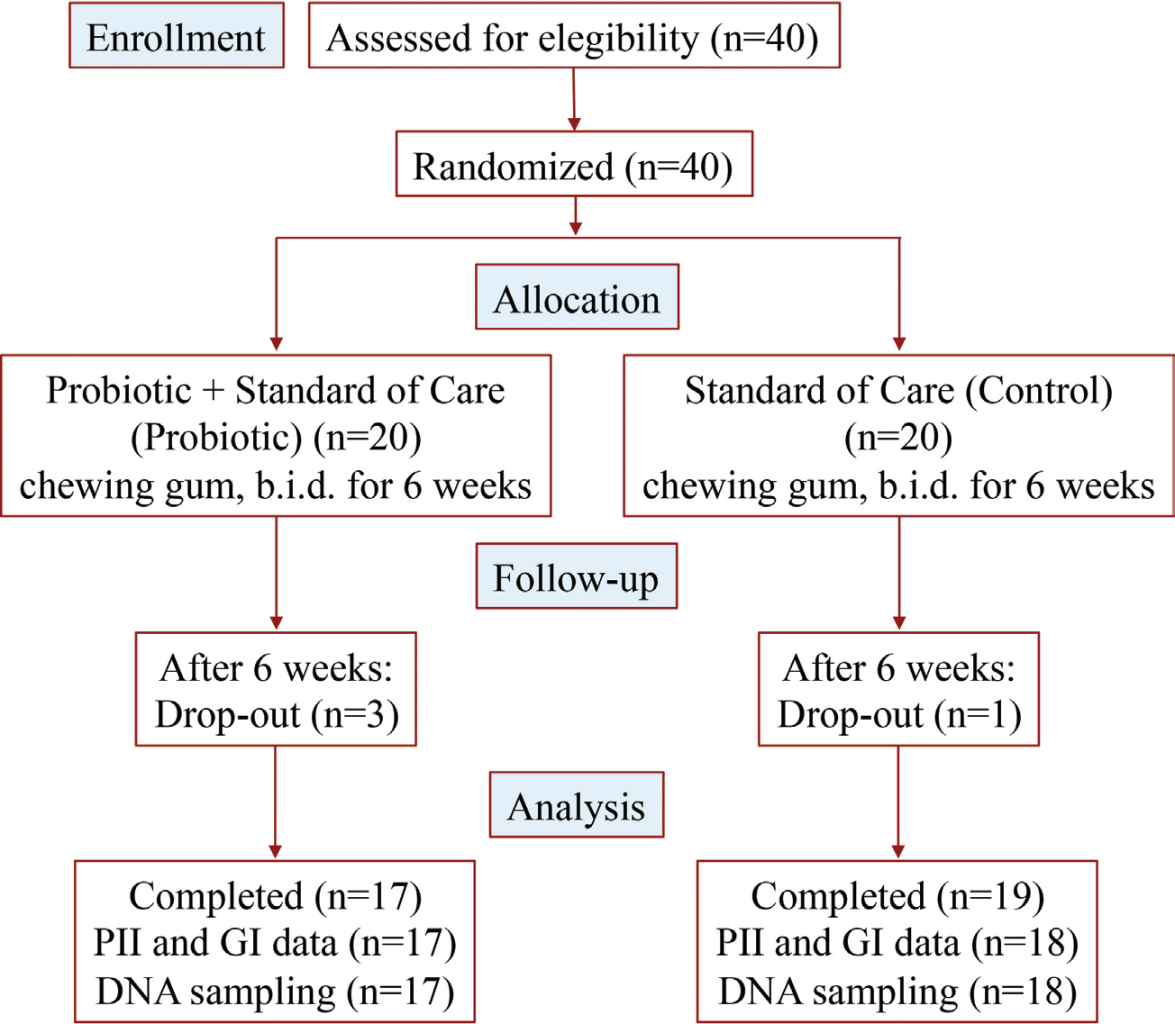
Participant CONSORT flowchart showing the number of participants who were randomly assigned, received intended treatment and were analysed.

The study was designed as a randomized, double-blind, placebo-controlled, 6-week intervention clinical trial. Volunteers were randomly allocated through a computer-generated random list and received coded product boxes containing blisters of either probiotic or placebo chewing gums accordingly. Identical chewing gums in identical blisters containing the allocated treatment (probiotic or placebo) were produced and coded by AB-Biotics SA (Barcelona, Spain), and the code was delivered to periodontics specialists after the last volunteer left the study. Therefore, both volunteers and periodontics specialists were blinded to the actual treatment given to each volunteer. A single examiner collected the following parameters at baseline and at the end of the 6-week intervention: saliva sample to assess probiotic colonization, gingival index (GI), plaque index (PII) and dental probing pocket depth (PD). A CONSORT flow chart explaining the design of the study is presented in Figure 1.

-Treatments

Volunteers had to take two chewing gums a day, in separate moments of the day, for a total of 6 weeks. Chewing gums had to be taken at least 1 hour after the previous meal and 1 hour before the following meal and had to be chewed for at least 15-20 minutes each. Probiotic chewing gums contained two different strains of lactobacillus, *L. brevis* KABPTM-052 (CECT7480) and *L. plantarum* KABPTM-051 (CECT7481), at a minimum dose of 5x108 CFU per chewing gum. The proportion of the strains was (1:1). Live bacterial load in the chewing gums (CFUs) was validated by plating 10-fold serial dilutions onto MRS agar and incubation in a microaerophilic environment at 37ºC for 72 h. Placebo chewing gums were indistinguishable in form, colour and taste to the probiotic chewing gums. Besides, the same fluorinated toothpaste (1,450 ppm) was given to all volunteers. All volunteers were instructed to use it twice daily during the study, as well as how to properly brush their teeth.

-Efficacy assessment

The primary efficacy endpoint was the colonization of the buccal cavity by *L. plantarum* KABPTM-051 (CECT7481) and *L. brevis* KABPTM-052 (CECT7480) at the end of the 6-week study period and was assessed by quantitative real-time PCR (qPCR). Buccal microbiota samples were collected at baseline before starting the chewing gum treatment, and at least 12 h after taking the last chewing gum after 6 weeks of therapy. One unstimulated saliva sample (0.5 ml) was taken from each volunteer and collected in 1.5 ml tubes and stored at −80°C prior to bacterial quantification. Volunteers did not ingest any food during 2 h or brushed their teeth during 6 h prior to sample collection.

The Community Periodontal Index (CPI) dental pieces (i.e. 11, 16/17, 26/27, 31, 36/37 and 46/47)(21) and tongue were considered for the specific assessment of the progression of Gingival Index (GI), Plaque Index (PII) and dental probing pocket depth (PD) as measured with a CP10 probe (Hu-Friedy, Germany).

-DNA isolation and qPCR

Samples were transported to the laboratory in dry ice and immediately sonicated for 42 seconds five times before centrifugation for 20 min at 10,000 g and 4°C. DNA was extracted using QIAamp DNA Blood Mini Kit (Qiagen, Spain) following manufacturer’s instructions.

Quantitative polymerase chain reaction (qPCR) was used to detect and quantify DNA of *L. plantarum* KABPTM-051 (CECT7481) and *L. brevis* KABPTM-052 (CECT7480). The qPCR amplification was performed with PowerUp SYBR Green Master Mix (Thermo Fisher Scientific, Spain) using specific primers (Biomers, Germany). For *L. brevis*, a 165 bp fragment of hsp60 gene was amplified using primers 5’-GCACAAGATGGCTCATGACGTTAAGACTAAGG-3’ and 5’-GTCTAAGCTCGTATCAACCCCACGGG-3’ (22). The primers used to amplify a 54 bp fragment of 16S gene of L. plantarum were 5’-CTCTGGTATTGATTGGTGCTTGCAT-3’ and 5’-GTTCGCCACTCACTCAAATGTAAA-3’ (23). Each sample was run in duplicate: 40 ng of sample DNA with 0.5 mM primers in a final volume of 10 μL. The qPCR conditions were as follows: 95 °C for 7 min, (95 °C for 15 sec, 64 °C for 1 min, 72 °C for 1 min) x 40 cycles. Quantification cycle (Cq) values, the PCR cycle number at which fluorescence rises above the baseline, were determined using the 7500 software v2.0.5 (Applied Biosystems). The correlation between Cq values and CFU/μg DNA was based on standard curves constructed with 10-fold serial dilutions of each bacterial DNA, from 108 CFU/μg to 102 CFU/μg DNA. All assays were developed with a linear quantitative detection established by the slope of 3.39 and 3.54 cycles/log decade, r2 of 0.999 and 1.000, and an efficiency of 97.22 % and 91.51 % for *L. brevis* KABPTM-051 and *L. plantarum* KABPTM-052, respectively. Measures to avoid carryover DNA were established.

-Compliance, product satisfaction and safety

Empty blisters returned by volunteers were counted to confirm treatment compliance. Product satisfaction was rated using Likert scales ranging 1 to 9 for taste, aroma, aftertaste, and global evaluation. Adverse events were monitored following the directives of the Spanish Pharmacovigilance System for standard clinical trials with drugs.

-Statistical analysis

Prior data on the *in vivo* colonization capacity of the strains under study was not available at the time of protocol design to undertake a sample size calculation. Therefore, we designed this study with an arbitrary sample size of 40 patients (20 per group).

Statistical analysis of probiotic colonization was performed on the population that completed the study. For baseline data, between-group comparisons were performed with Student T-test for quantitative variables and Fisher’s exact test for dichotomous variables. Bacterial concentration data (copies of DNA) corresponding to baseline and end of intervention was normalized using a log transform. Change in bacterial concentration between groups was assessed using a repeated measures general linear model. The same approach was used to assess change in plaque index (PII), gingival index (GI) and pocked depth (PD). No clustering of samples was performed in repeated measures analysis because a single value was analysed per subject and timepoint. Product satisfaction ratings were analysed by means of Mann-Whitney test. Finally, correlation between log-transformed DNA copies and change in PII, GI and PD were tested using Spearman´s rank test, and changes within group in the proportion of subjects with dental plaque were assessed with the exact version of McNemar’s test (Lidell’s test). All statistical analyses were performed with SPSS (v.20.0, IBM Corporation), and significance threshold was set at two-sided *p* = 0.05.

## Results

-Study Population

A total of 40 volunteers (20 per group; age range 18-55 years) were enrolled in the study, and 4 of them (10%, 3 in probiotic group and 1 in placebo group, *p* = 0.605) dropped out from the study and did not attend the follow-up visit. All volunteers had mean gingival index (GI) in the 0 to 1.1 range (i.e. healthy to mild gingivitis), brushed their teeth twice daily on average, and none of them had dental pockets more than 3 mm deep. Overall, there were no statistically significant differences between groups in none of the measured parameters at baseline, although the proportion of women to men was noticeably higher in probiotic group and GI was somewhat higher in placebo group (Table 1). Of note, both *L. brevis* and *L. plantarum* were detected in the study subjects at baseline, with abundance of L. brevis (log of CFUs per μg of DNA) being lower than that of *L. plantarum*. This observation indicates these bacteria can be found in the oral cavity of healthy subjects.

**Table 1:**
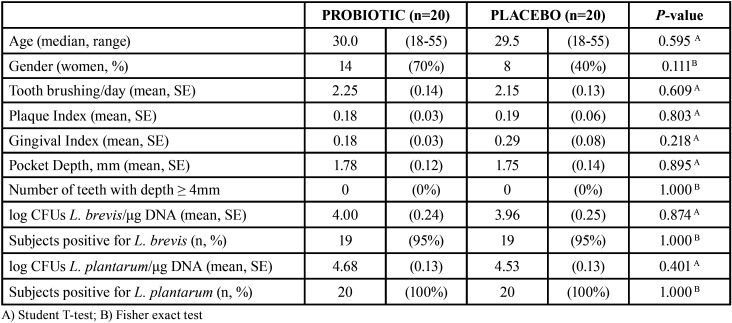
Baseline data of enrolled subjects.

-Colonization by *L. brevis* KABPTM-052 and *L. plantarum* KABPTM-051

**Figure 2:**
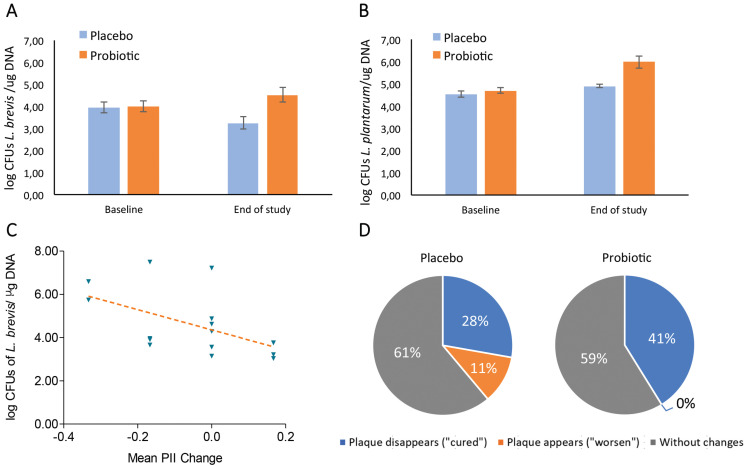
Colonization of AB-Dentalac strains and effect on evolution of dental plaque. A: Mean and SE of *L. brevis* at baseline and end of study from buccal samples of volunteers treated with probiotic (orange bars) or placebo (blue bars), repeated measures *p*=0.017 for probiotic vs placebo. B: Mean and SE of *L. plantarum* at baseline and end of study from buccal samples of volunteers treated with probiotic or placebo, repeated measures *p* = 0.004 for probiotic vs placebo. C: Correlation between dental plaque change (mean PII change) and levels of *L. brevis* (Log of DNA copies). Spearman rho = 0.57; *p* = 0.022. D: Evolution of dental plaque measured as the percentage of subjects “cured”, “worsen” or “without changes” in placebo and probiotic groups; 41% net reduction of plaque in probiotic group, in 7 out of 17 volunteers (*p* = 0.016).

Repeated measures analysis indicated both *L. brevis* and *L. plantarum* significantly increased throughout the study in the probiotic-supplemented group compared to placebo (*p* = 0.017 and *p* = 0.004, respectively). Significance was maintained after adjusting the repeated measures model for the variables displaying the largest imbalance at baseline between groups, both by considering gender only and by considering gender and baseline gingival index. At the end of the study (week 6), mean concentrations of *L. brevis* and *L. plantarum* were 1 order of magnitude (i.e. 10 times) higher in probiotic group than in the placebo one (Fig. 2A,B).

-Correlation between gingival health and *Lactobacillus* levels

As per entry criteria, none of the subjects displayed a clinically relevant gingivitis or pocket depth, and no significant differences between probiotic and placebo were detected in PII, GI and PD throughout the study, as expected (data not shown). However, reduction in mean PII was significantly correlated to higher levels of *L. brevis* at the end of the intervention in probiotic group (Spearman rho = 0.57; *p* = 0.022; Fig. 2C), but not in the placebo group. In this regard, 16 subjects in the probiotic group displayed dental plaque at baseline in one or more of the predefined CPI teeth, compared to 9 at study endpoint without any case of de novo plaque appearance (*p* = 0.016 for the reduction). Conversely, 11 subjects in the placebo group had dental plaque at baseline among the predefined teeth, compared to 8 at study endpoint, where dental plaque disappeared in 5 volunteers and 2 other subjects had de novo plaque (*p* > 0.10 for the reduction) (Fig. 2D). No other significant correlations were detected between mean dental indices and concentration of *L. brevis* and *L. plantarum*.

-Compliance, product satisfaction and safety

Treatment product and placebo were equally well tolerated by all volunteers and no adverse effects were recorded. Only 10 % of subjects dropped-out during the study period, 3 in the probiotic group and 1 in the placebo group (a non-significant difference). Treatment compliance was very high (99%), without differences between group. No differences were found in the global evaluation of the product, texture rating and aftertaste rating, but a trend for lower taste with probiotic was observed in comparison to placebo chewing gums (*p* = 0.093). No adverse reactions were reported during the study.

## Discussion

Previous studies have suggested a beneficial role of low acidogenic probiotic strains in colonizing buccal cavity to displace oral pathogens (3,7), although evidence remains inconclusive regarding the choice of best strains. In this regard, Montero and colleagues (5) demonstrated that 6-week consumption of a probiotic formula containing *Levilactobacillus brevis* KABPTM-052 (CECT7480) and *Lactiplantibacillus plantarum* KABPTM-051 (CECT7481), together with *Pediococcus acidilactici* KABPTM-053 (CECT8633), was able to reduce the counts of the periodontopathogen *Tannerella forsythia* in subjects with gingivitis. Similarly, Ferres-Amat and colleagues also demonstrated a reduction in postoperative pain against placebo when administering *L. brevis* KABPTM-052 and *L. plantarum* KABPTM-051 to subjects undergoing mandibular third molar extraction. Other studies have shown that the treatment with different probiotic strains can help reduce specific pathogens like *Streptococcus mutans* (24), **Prevotella intermedia** (25) and **Porphyromonas gingivalis** (26), as well as the reduction of caries (27).

Despite the above-mentioned effects of some probiotics in the oral microbiome, it stills to be proved whether these probiotic strains build-up in the oral microbiota. The results of this randomized, double-blinded, placebo-controlled clinical trial show that 6-week supplementation with probiotic strains *L. brevis* KABPTM-052 and *L. plantarum* KABPTM-051 significantly increased the concentration of L. brevis and *L. plantarum* by 1 order of magnitude (1 log) compared to placebo. Of note, both species were found in the oral microbiota of healthy volunteers, as demonstrated by the baseline levels of both lactobacilli in our study. Tooth brushing habits at baseline were identical between groups, all volunteers used the same fluorinated toothpaste during the study and the use of mouthwashes was controlled, further supporting the hypothesis that the differences in the abundance of both *L. brevis* and *L. plantarum* were due to the specific probiotic intervention and not to random external factors. Moreover, the significance of the effect against placebo was maintained when adjusting by gender and baseline gingival index, which displayed some imbalance at baseline between groups. This study also confirmed that this probiotic was well tolerated by the volunteers and that no adverse effects were reported in the treatment group or placebo group.

This study aimed to assess probiotic colonization in subjects with healthy gingiva or mild gingivitis, as a first step towards demonstrating their usefulness as a preventive therapy. Accordingly, gingival index, plaque index and probing pocket depth were mild at baseline, and no significant differences between groups were noted in the evolution of said indexes during the intervention, as expected. However, an exploratory analysis found oral cavity colonization by this probiotic was positively correlated with a reduction in mean plaque index. Moreover, the number of subjects displaying dental plaque was significantly reduced by 41 % in probiotic group compared to baseline, but no statistically significant difference was observed in placebo group. Previous studies found that *L. brevis* had a higher affinity for hydroxyapatite teeth surface than *L. plantarum* (28). These results could explain the observed correlation between a reduction in mean plaque index and higher concentration of *L. brevis* but not of *L. plantarum* at study endpoint, as the volunteers who displayed the largest reduction in mean plaque index were the ones with higher colonization of *L. brevis* KABPTM-052 (CECT7480) at study endpoint.

One limitation of this study is that microbiota was investigated from saliva samples by qPCR analysis. This sampling methodology did not allow for a confirmation of the tissue-specific adhesion of each strain as reported *in vitro* (18). In addition, qPCR quantification didn’t let us to discriminate between live and dead bacteria. However, since that the last chewing gum was consumed by volunteers between 12-36 h before the sampling procedure, it is unlikely free DNA could cause the significant increment of *L. plantarum* and L. brevis DNA in probiotic due to the continuous wash-out effect of saliva. Moreover, the association between reduced plaque index and increased levels of *L. brevis* leads us to hypothesize that active *L. brevis* was effectively colonizing oral surfaces of volunteers, in line with previous *in vitro* findings of *L. brevis* high adherence to teeth (28). Another limitation is that qPCR primers were species-specific and amplification of other strains of species L. brevis and *L. plantarum* cannot be ruled out. In fact, significant detection of both *L. brevis* and *L. plantarum* at baseline indicates strains of these species were common in the oral microbiota of the study volunteers. Nevertheless, because of the randomized, placebo-controlled design, lack of differences at baseline between groups, and standardization of oral hygiene habits during the study, the significant increment of the abundance of both *L. brevis* and *L. plantarum* species in the probiotic-treated group can be attributed to the specific supplementation with strains *L. plantarum* KABPTM-051 and *L. brevis* KABPTM-052.

In summary, *L. plantarum* and *L. brevis* were detected in the saliva of health volunteers in this pilot study, and a 6-week administration of probiotic chewing gums led to a significant increase in both *L. plantarum* and *L. brevis* compared to placebo, the difference averaging 1 order of magnitude. As expected, inclusion of healthy subjects prevented the observation of significant effect on plaque and gingival indexes compared to placebo. However, an exploratory analysis identified a reduction in number of subjects with dental plaque in the probiotic group but not the placebo one. In our view, this finding warrants additional studies with a larger sample size to confirm the effect of these specific probiotic strains on dental plaque build-up. Such effect could be of interest as an adjunctive preventive treatment for gingivitis, by means of reducing plaque formation. Moreover, to gain insight in the strain’s mechanism of action, analysis by metagenomic techniques of the complete oral microbiota at various buccal sites pre- and post-intervention would be needed.
